# A Unique Case of Pyruvate Carboxylase Deficiency

**DOI:** 10.7759/cureus.15042

**Published:** 2021-05-15

**Authors:** Jessica Hidalgo, Leticia Campoverde, Juan Fernando Ortiz, Samir Ruxmohan, Ahmed Eissa-Garcés

**Affiliations:** 1 Internal Medicine, Universidad San Francisco de Quito, Quito, ECU; 2 Oncology, Bethesda Hospital East, Boston, USA; 3 Neurology, Universidad San Francisco de Quito, Quito, ECU; 4 Neurology, Larkin Community Hospital, Miami, USA

**Keywords:** pyruvate carboxylase, nervous system, seizures, infantile spasm, lactic acidosis

## Abstract

Pyruvate carboxylase (PC) converts pyruvate to oxaloacetate, which is an important step in gluconeogenesis. Pyruvate carboxylase deficiency (PCD) is a rare inherited metabolic disorder characterized by movement disorders, neurologic disturbances, hypoglycemia, lactic acidosis, hyperammonemia, and elevated levels of pyruvate and alanine in plasma. The prognosis for PCD is poor. Most children die within the first six months of life, and those who survive longer have neurological damage and mental disability. This is due to the accumulation of lactic acid and toxic components in the blood. Here we describe the case of a 21-month-old male presenting with abnormal movements and new-onset seizures. His family history is relevant because of parental consanguinity. A genetic analysis showed a novel mutation, homozygous c. 2630A>G (p. Gln877Arg) variant, in the PC gene, a mutation not previously described in the English literature.

## Introduction

Pyruvate carboxylase (PC) is a metabolic enzyme that catalyzes the irreversible carboxylation of pyruvate into oxaloacetate [1]. Pyruvate carboxylase deficiency (PCD) is a rare autosomal recessive metabolic disease with an estimated incidence of one in 250,000 births, resulting in abnormally high pyruvate, lactic acid, and alanine levels [2-3]. This inherited disorder is caused by a mutation in the PC gene, which results in the accumulation of toxic substances in the blood, causing multiorgan damage, especially in the nervous system [2]. This disease can cause failure to thrive, developmental delays, motor disturbance, mental and growth retardation, seizures, and lactic acidosis starting in the neonatal or early infantile period [2,4].

PCD is divided into three types, depending on the severity of signs and symptoms [4]. PCD type A, also called the North American type, is characterized by severe symptoms that begin in infancy. Features include developmental delay, hypoglycemia, and lactic acidosis, which causes vomiting, abdominal pain, muscle weakness, and fatigue [5]. Type B, also called neonatal or French form, usually presents within the first 72 hours of life. This type is characterized by severe lactic acidosis, hyperammonemia, truncal hypotonia, coma, and liver failure [5]. Type C, the benign variety, is extremely rare, and it is characterized by a normal or slight increase in lactic acid levels, with little impact on the nervous system [2,6].

Here we present a case of PCD type A presenting with tonic-clonic seizures, infantile spasms, chorea-like movements, and metabolic acidosis. The case is novel and unique, as it is a variant of a gene that has not been described before. We aim to compare this case to the standard presentation of PCD type A and explain the pathophysiology related to the patient's clinical features.

## Case presentation

History of present illness

A 21-month-old male was brought to the emergency department by his mother due to abnormal movements for one month. These episodes were described as tilting of the head to the left side, left gaze deviation, and fencer posturing toward the left for a few seconds with clusters up to two to three minutes. Additionally, the mother reported that the child had sudden, jerky, purposeless movements in the upper extremities. A video electroencephalogram (EEG) showed epileptic spasms, tonic-clonic seizures, and multifocal epileptiform discharges; findings consistent with epileptic encephalopathy and infantile spasms.

Past medical history

This patient was diagnosed with PCD as a neonate. The newborn hearing screening was abnormal, suggestive of auditory neuropathy spectrum disorder. On the fifth day of birth, the patient presented respiratory distress, spasticity, hypertonia, and abnormal body movements.

Physical exam

Table 1 shows the initial physical exam of the patient on admission to the hospital.

**Table 1:** Physical exam of the patient HEENT: head, eyes, ears, nose, and throat; Kg: kilograms

Physical Exam			
Weight	11.45 kg	HEENT	Normocephalic. Conjunctivae and lids normal. Pupils round, symmetric, reactive to light and accommodation. Pinnae and nose without trauma. Neck supple.
Constitutional	Afebrile. Appears well-nourished, with no acute distress.	Musculoskeletal	Digits and nails unremarkable. Occasional contractions and spasms of the upper extremities and left torso.
Cardiopulmonary	No extremity edema, murmurs, or pathological pulmonary sounds.	Gastrointestinal-renal	No abnormalities, no masses, no tenderness.
Skin	No visible or palpable lesions. There are no cutaneous stigmata of neurological disease.	Psychiatric	Global delay.
Neurological Exam			
Cranial nerves	Pupils pinpoint sluggishly reactive to light bilaterally. Extraocular movements grossly intact, face movements symmetric, tongue appears midline.	Motor	Spontaneous movements in the four extremities, normal bulk, axial hypotonia with increased appendicular tone. Chorea-like movements in the right upper extremity.
Sensory	Responds to tactile stimuli, withdrawals to tactile and deep nailbed pressure.	Reflexes	1+ bilaterally at biceps, brachial, knee, ankle. Induced clonus presents bilaterally in the ankles.
Developmental History			
Gross motor	Rolling over, poor head holding, not sitting on his own.	Speech	Babbles, no words. As per parents, turns to sound.
Social	Social smile, does not play with toys	Fine motor	Cannot reach out. Cannot transfer an object from one hand to another.

Blood tests

All the results were normal, except for an elevated lactic acid (3.5 mmol/L) and a lactic acid/pyruvate ratio of 19. For this reason, an organic urine analysis was ordered, and the results showed markedly elevated levels of lactic, 3-hydroxybutyric, acetoacetic, 4-hydroxyphenyl acetic, and 4 hydroxyphenyl pyruvic acids. A peracetic acid test (PPA) was done, and it showed that methionine, valine, tyrosine, and citrulline were elevated.

Magnetic resonance imaging

**Figure 1:**
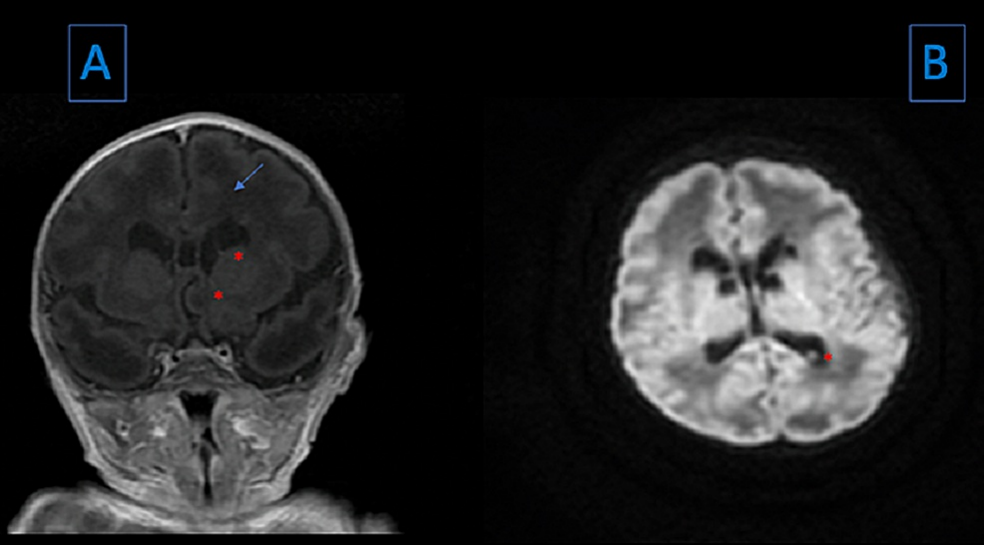
MRI findings Image A: MRI T1 sequence, coronal view. The blue line shows an enlargement of the lateral ventricles, the red asterisks show multiple periventricular/subependymal cysts in the caudothalamic grooves; Image B: MRI T2 FLAIR, sagittal view: the red asterisks show a subependymal cyst. MRI: magnetic resonance imaging; FLAIR: fluid-attenuated inversion recovery

Prominent lateral ventricles, with apparent multiple septations mainly anteriorly. There were multiple periventricular/subependymal cysts in the caudothalamic grooves next to the temporal posterior and frontal horns and body of the lateral ventricles. Figure 1 shows the main MRI findings of the case.

Electroencephalogram

A 24-hours electroencephalogram (EEG) study during sleep showed shifting slow and occasional sharp waves occurring independently over both temporal regions. These findings indicate potentially epileptogenic dysfunction.

Genetic testing

The abnormal chromosomal microarray test showed multiple areas of homozygosity totally approximately 398 MB, + Parental Consanguinity - 1st cousins,

Final rapid whole-genome sequencing showed a homozygous c. 2630A>G (p. Gln877Arg) variant in the PC gen. Table 2 shows the biparental genome of the patient.

**Table 2:** Biparental genome shows variants of unknown significance to the patient's phenotype

Gene (Transcript)	Condition	Chromosome: Genomic Coordinates	Variant	Zygosity (Inheritance)	Classification
PC (ENS0000393960)	Pyruvate carboxylase deficiency	11:6617779	c.2630A>G (p.Gln877Arg)	Homozygous (Biparental)	Uncertain significance

Impression

This variant has not been previously reported or functionally characterized in the literature to our knowledge. It is absent from the Exome Aggregation Consortium (ExAC) and Genome Aggregation Database (gnomAD) population databases, and this is presumed to be rare.

Impression and treatment

The patient was diagnosed with new-onset infantile spasms and tonic-clonic seizures, which were expected due to metabolic disorder, PCD type A. A genetic test revealed a variant of uncertain significance that has not been described before to our knowledge.

Topiramate was added to decrease the frequency of the seizures. While this medication was effective at the beginning, the treatment was discontinued because of worsening lactic acidosis. Clobazam was added and well-tolerated. The frequency and dose of prednisone were increased to manage the infantile spasms.

A high carbohydrate and protein diet was recommended. The patient was discharged with the following medications: thiamine 50 mg BID, aspartic acid 28 mcg QD, biotin 5000 mcg QD, citric acid-sodium citrate 20 ml QID, clobazam 2.5 mg BID, triheptanoin 8 ml BID, levocarnitine 250 mg BID, prednisolone 30 mg TID, soybean oil 5 ml QD.

At the one-month follow-up, the mother reported a decrease in the frequency of the seizures, chorea-like movements, and spasms. The prognosis of the patient is unknown mainly due to the natural course of the disease and the limited literature regarding PCD type A.

## Discussion

PCD is a rare neurometabolic disorder with less than 40 cases reported [7]. Our patient’s symptoms started in the first month of life, presentation consistent with PCD type A. Most children with PCD type A will die in infancy or early childhood. In contrast, most patients with type B will die in the neonatal period, and patients who survive remain unresponsive and die due to respiratory infections before five months of age. There are reports of patients who have survived until age nine and 20, respectively, probably because of mosaicism [8].

The most common differential diagnosis of PCD is pyruvate dehydrogenase complex (PDHC) deficiency, which is caused by the lack of one of the three catalytic components of the enzyme (E1, E2, and E3). Both PCD and PDHCD patients present with lactic acidemia and intermittent neurologic features such as hypotonia, seizures, loss of motor milestones, ataxia, and episodic dystonia. There is no clinical difference between PCD and PDHCD. High levels of lactate can be found in the cerebrospinal fluid and are associated with pyruvate and alanine elevation. Hypoglycemia caused by fasting can be present because of increased pyruvate carboxylation and gluconeogenesis [8]. One of the differences is that blood ketone bodies are not detectable in PDHCD, unlikely PCD. Also, PDHCD presents with a normal lactate/pyruvate ratio in plasma as compared to PCD in which it is increased [4]. Macrocephaly may be a prominent feature of PCD; although this feature is often reported, the exact incidence is unclear due to the frequent lack of head circumference measurement data [9].

Sudden, jerky, purposeless movements have been described in patients with PCD [9]. The patient had a chorea-like movement, which can be associated with a cyst in the caudothalamic grooves seen in the brain MRI.

Metabolic findings in this disorder are the elevation of lactic acid, alanine, and pyruvate in the blood. The lack of pyruvate carboxylase causes the accumulation of high levels of pyruvate, which are converted into lactic acid by lactate dehydrogenase enzyme, leading to an increased lactic acid concentration in plasma [7]. Lactic acid levels vary depending on the type of PCD. Common ranges are: 2-10 mmol/L for type A, >10 mmol/L for type B, and 2-5 mmol/L for type C. The lactate/pyruvate ratio in type B is elevated (>20) while type A and C usually are normal (<20) [2,4]. In our patient, the lactic acid levels were between 3.5 mmol/L to 8.5 mmol/L, and the lactate/pyruvate ratio was 19, consistent with PCD type A.

The treatment for this condition focuses on providing alternative energy sources, hydration, and metabolic acidosis correction in acute episodes of decompensation. The therapy includes pharmacological doses of co-factors involved in the metabolism of pyruvate and the substitution of the missing end-products [9]. Aspartic acid is needed because in PCD, oxaloacetate biosynthesis and the Krebs cycle are impaired. Depletion of aspartate disrupts the urea cycle; therefore, it is necessary for patients to receive aspartic acid [10]. Lipoic acid and thiamine (coenzymes for pyruvate dehydrogenase), biotin (regulator of pyruvate carboxylase activity), and citrate (which reduces acidosis and provides the substrate in the citric acid cycle) are also needed [9,11]. Aspartic acid lowers ammonia levels, letting the urea cycle take place [12]. Lorazepam and clobazam are used to treat seizures in patients with PCD, and prednisone controls infantile spasms [13]. The treatment that our patient received was: thiamine, aspartic acid, biotin, citric acid-sodium citrate, clobazam, and levocarnitine, which is appropriate according to the literature [12].

Our patient had a variant mutation in the pyruvate carboxylase gene c. 2630A>G (p.Gln877Arg), which has not been reported in the literature before, so the severity and prognosis of this disorder in this patient are unknown. For this reason, frequent follow-up is recommended.

## Conclusions

Pyruvate carboxylase deficiency is a rare metabolic disorder with just a few cases reported, so it is essential to report this patient’s case. This patient had chorea-like movements, possibly caused by caudothalamic groove cysts seen in the brain MRI. The infantile spasms and seizures seen in this patient are associated with metabolic imbalance disorder. The frequency of the seizures, spasms, and chorea-like movements improved with clobazam and prednisolone. Also, it was important to correct the biochemical imbalance to enhance the patient’s symptoms. The variant mutation in the PC gene reported in this patient has an uncertain significance, which has not been reported in the literature before, so the prognosis and severity of this disorder are unknown. More cases of PCD type A need to be described in the literature to consolidate the knowledge of this disorder.
